# The experience of long-stay patients in a forensic psychiatric hospital in China: a qualitative study

**DOI:** 10.1186/s12913-019-4458-6

**Published:** 2019-09-02

**Authors:** Shaoling Zhong, Huijuan Guo, Yuanyuan Wang, Stephanie Cook, Yanan Chen, Chenyuli Luo, Ke Peng, Fanglan Wang, Xiaoxi Liang, Hui Chen, Qiguang Li, Jiansong Zhou, Xiaoping Wang, Runsen Chen

**Affiliations:** 1 Department of Psychiatry of the Second Xiangya Hospital, Central South University, China National Clinical Research Center on Mental Disorders; China National Technology Institute on Mental Disorders; Hunan Key Laboratory of Psychiatry and Mental Health, Mental Health Institute of Central South University, Changsha, 410011 Hunan China; 2 Division of Psychology, Faculty of Health and Life Sciences, De Montfort University, Leicester, UK; 3 The George Institute for Global Health, UNSW, Sydney, Australia; 4 School of Public Health, The University of Sydney, Sydney, Australia; 5 The National Clinical Research Center for Mental Disorders & Beijing Key Laboratory of Mental Disorders, Beijing Anding Hospital, Capital Medical University, Beijing, China; 6 Department of Psychiatry, University of Oxford, Oxford, UK

**Keywords:** Long-stay, Forensic, Psychiatric, Qualitative, Crime

## Abstract

**Background:**

Long stay in forensic psychiatric hospitals is common in patients who are defined as “not criminally responsible on account of mental disorder”. However, little is known about how these patients experience and perceive the long stay within these settings. The aim of this study is to explore the perception and needs of long-stay patients in forensic psychiatric hospitals in China.

**Methods:**

In-depth semi-structured interviews were conducted with 21 participants who had lived in the forensic psychiatry hospital for more than 8 years. We used thematic analysis strategies to analyse the qualitative data.

**Results:**

Participants’ perceptions clustered seven themes: hopelessness, loneliness, worthlessness, low mood, sleep disturbances, lack of freedom, and lack of mental health intervention.

**Conclusions:**

The views and opinions expressed by long-stay patients showed that psychological distress is prevailing in forensic psychiatric hospitals. Adequate and effective care and mental health interventions are recommended to be tailored for their special needs.

**Electronic supplementary material:**

The online version of this article (10.1186/s12913-019-4458-6) contains supplementary material, which is available to authorized users.

## Background

Mentally disordered offenders in China who are defined as “not criminally responsible on account of mental disorder” are given mandatory treatment by the government in forensic psychiatric hospitals (Ankang hospitals), which are similar to the medium and high security hospitals in the United Kingdom and the maximum security hospitals in North America [1]. The aim of the detention is to improve the mental health status and also reduce the risk of violence in these patients [2]. It was reported that there were more than 22 forensic psychiatric hospitals holding more than 7000 patients in China [1]. Compared to Western countries, the increasing rate of forensic psychiatric beds in China is modest, with an average of 1.1% between 1990 and 2009 [3]. Nevertheless, such an increase comes at a high cost, both financially and in terms of resourcing.

Studies have consistently reported that long stay in forensic psychiatric hospitals is common in many countries [4, 5]. As there is no specific threshold defining long-stay, this varied across studies, ranging from 2 to 10 years [6–8]. For example, in England, a recent study reported that around 16% of patients stayed for more than 10 years, and 3% stayed for more than 20 years in high security settings [9]. Although differences in legislation and health care settings may hamper the international comparisons of length of stay, quantitative studies have identified profiles of demographics (e.g. male, unmarried), criminal history (e.g. history of violence, murder or homicide as index offence) and clinical factors (e.g. multiple previous inpatient admissions and risk of absconding) associated with longer stay [4, 6, 10–12]. Furthermore, there are concerns that some patients do not experience adequate levels of security care [7, 13], while the quality of life in forensic services may be poor [14].

Evidence suggests that long-stay patients in forensic settings have special needs for maintaining quality of life alongside treatment for mental disorder, including daytime activities improving social skills, self-esteem and physical health, and addressing communication difficulties [15, 16]. Before improving health-care and developing intervention programs to address the needs, the experience of the long-stay patients should be established. A significant number of negative emotions and experience, including but not limited to loneliness [17], hopeless [18] and stigma [19] were identified in patients in previous studies. However, little is known about how mentally ill offenders experience and perceive long stay in forensic psychiatric hospitals in China.

Understanding the perceptions of long stay patients in forensic psychiatric hospitals is essential to maximizing the resources of, whilst minimizing the cost to mental health services in addressing risk reduction and relapse prevention. With the complexities of mental disorders and violent behavior taken into consideration, qualitative methods could enhance the richness of the data and the overall quality of the research [20]. Therefore, the aim of this study is to investigate mentally ill offenders’ experiences and perceptions in relation to long stay at a forensic psychiatric institution using qualitative methods.

## Methods

### Participants

This study was approved by the Human Ethics Committee of the Second Xiangya Hospital of Central South University**.** Participants were selected from Hunan Province Forensic Psychiatry Hospital in China using a purposive sampling approach. We asked the hospital’s psychiastrists to identify volunteers to participate in our study. Each of the participants were given a letter outlining the purpose of the study and a consent from. The Forensic Psychiatry Hospital is located in Yueyang, Hunan province, in the central-South of China, which has a population of 67 million. It is the only forensic psychiatry hospital of the province, equipped with five wards, 13 clinicians and 21 nurses. There were 461 mentally ill offenders, the majority of whom were diagnosed with *schizophrenia* (90.5%) in the hospital at the time of investigation. The average length of stay was 8.0 ± 4.8 years, and the 45.6% of the total sample had stayed for more than 8 years. We used the average length of stay of the whole sample as a cut off score for the inclusion criteraion. Therefore, participants were required to have lived in the hospital for at least 8 years (long-stay criteria) and to be currently diagnosed with a mental illness. Participants were required to be in a stable condition with good insight (based on their psychaitrist’s assessment), and were approved to take part in the study by their psychiatrist. Participants were all able to communicate in Mandarin.

### Procedure

Participants were invited to take part in individual semi-structured interviews. The interview was designed to capture the patients’ experience and perception of the forensic psychiatry hospital. A comprehensive interview schedule was created via a literature review carried out by two Chinese forensic psychiatry experts. We searched Chinese and English language articles using the search terms “forensic hospital”, “long-stay”, “mental health”, “offenders”, “secure hospital”, “mental illness”, “reoffend” and “quality of life” using Google Scholar, PubMed, PsycINFO, Wanfang and Zhiwang. However, we did not identify any previous studies that had been conducted on mentally ill offenders’ experiences and perceptions of long stay in forensic psychiatric hospitals in China. The interview schedule was developed based on previous qualitative studies from other countries (see Additional file 1) [18, 21–26]. All interviews were conducted in a private meeting room in the hospital by graduate students studying forensic psychiatry. The three interviewers had received relevant training and also had access to ongoing mentoring for interview skills and research ethics. Each interview lasted approximately 40 min to 1 hour.

### Data analysis

All interviews were audio-recorded and then transcribed verbatim by a professional academic transcription company. The data were analyzed using a thematic analysis approach. The following five phases were included in our thematic analysis approach [27]: 1) two authors (CR and ZS) independently familiarized themselves with the raw data by reading and rereading the data while making notes on the initial interpretation; 2) coded each datum (label with a term indicating the interpretation); 3) collated codes into the potential themes; 4) reviewed the themes and checked the logic regarding the extracts and all dataset; 5) defined and named the final thems. Authors repeated the coding and identifcaiotn of themes in order to make sure the coding was consistent and that the themes were clearly defined. The two authors then held several meetings to discuss the themes they had generated in order to reach a consensus on the accuracy of the data interpretation. The two authors generated similar interpretations of the transcripts. This consensus strengthens the validity of the current findings [28]. Also, an experienced forensic psychiatrist (WX) read over the themes in order to assess the feasibility of our findings. We also conducted a participant validation process in order to ensure that the transcripts accurately reflected participants’ perceptions (member checking) [29]. We discussed the findings with two of the original participants to clarify the key points and themes to check the validity of our interpretations. These were two participants (one male and one female) who previously indicated their willignness to be involved in checking our interpretations. The two participants confirmed that the content reflected their perceptions and experiences of living in the forensic psychiatry hospital.

## Results

Twenty-one participants with an average age of 45 years old took part in the study. Most participants were men (*n* = 19), and all participants were diagnosed with *schizophrenia* (100%, *n* = 21). The average length of stay in the hospital was 13 years. See Table 1 for further participant demographics. The seven main themes that were key to understanding the experiences of living in the Chinese forensic psychiatric hospital for long-stay patients were hopelessness, loneliness, worthlessness, low mood, sleep disturbances, lack of freedom, and lack of mental health intervention.

**Table 1:** Participant characteristics

Participant No	Gender	Age	Length of stay (years)	Marital status	Diagnosis	Cause of case
1	Male	62	22	Divorced	*Schizophrenia*	Intentional homicide
2	Male	44	21	Unmarried	*Schizophrenia*	Intentional homicide
3	Male	38	13	Unmarried	*Schizophrenia*	Intentional homicide
4	Male	54	14	Divorced	*Schizophrenia*	Intentional homicide
5	Male	45	17	Unmarried	*Schizophrenia*	Intentional homicide
6	Male	34	8	Unmarried	*Schizophrenia*	Intentional homicide
7	Male	53	8	Married	*Schizophrenia*	Intentional homicide
8	Male	33	11	Unmarried	*Schizophrenia*	Intentional homicide
9	Male	36	12	Unmarried	*Schizophrenia*	Intentional homicide
10	Male	40	11	Unmarried	*Schizophrenia*	Intentional homicide
11	Male	55	33	Unmarried	*Schizophrenia*	Intentional injury
12	Male	53	9	Unmarried	*Schizophrenia*	Intentional homicide
13	Male	47	13	Unmarried	*Schizophrenia*	Intentional homicide
14	Male	47	8	Divorced	*Schizophrenia*	Intentional injury
15	Male	34	11	Unmarried	*Schizophrenia*	Intentional homicide
16	Male	52	8	Unmarried	*Schizophrenia*	Intentional homicide
17	Male	40	15	Unmarried	*Schizophrenia*	Intentional injury
18	Male	41	10	Unmarried	*Schizophrenia*	Intentional homicide
19	Male	34	9	Unmarried	*Schizophrenia*	Intentional homicide
20	Female	46	10	Married	*Schizophrenia*	Intentional homicide
21	Female	48	8	Divorced	*Schizophrenia*	Intentional injury

### Hopelessness

#### Hopelessness surrounding mental illness

Remission is based on the premise of recovery of insight, which also decides whether the patient is well enough to be discharged. Most participants acknowledged that their symptoms had improved after medical treatment in the hospital. However, many participants felt frustrated when informed that they needed further assessment for full recovery. They complained about the lack of communication with the doctors and other medical staff, and suggested that they had no solid understanding of the definition of full recovery. In fact, most participants were not aware of the name and effects of their medication on their current condition. Some complained that the doctors did not respond to their questions about medication.

*I feel that I am well now, just like normal people, but still need to take medication every day. I feel desperate about this.*

*The dose is according to the hospital’s rules. Once you are in hospital, you’re supposed to just listen to your doctors and take this.*

*Doctors take no questions. They say the prescription is not our business. Knowing more about the medication is no good for us; we are not doctors.*

#### Hopelessness about discharge rules

When asked about their viewpoints regarding the discharge rules, most participants were pessimistic about whether they would be discharged. Some even felt that they might be there forever. Participants did not know much about the legal processes of discharge and were only told to stay peacefully and follow the rules. They were keen to have more information about the legal processes.

*I might as well be kept here forever. I indeed murdered someone.*

*Long-stays take away our hope. This may have a bad effect on the disease because we can’t stop thinking about getting out.*

*The discharge rules should be explicit. Ambiguity is no good for patients.*

### Loneliness

Many participants reported loneliness during their long stay. Contact with family can provide relief, but some participants had no parents to talk to. A lack of social support may aggravate their loneliness. Some participants suggested that their depressive feelings improved after talking to their family. In comparison to phone calls, family visiting was rare. Most family members only visited once a year.

As the law states that family members should serve as the guardians for formally admitted patients after discharge, whether the recovered patients can be discharged depends largely on their family members’ decisions. However, many patients were not in contact with their families. Moreover, some patients suggested that their family refused to accept them. Some felt upset and hopeless that their family did not talk to them about discharge.

During holiday seasons, the hospital would hold some celebrations. Participants had mixed feelings about holidays. On the one hand, they were happy about the celebrations; on the other hand, they would miss their families. They hoped that family visits would be allowed during holidays in order to offer some emotional support.

*It (the stay) is too long. I feel extremely lonely and like there’s no hope at all.*

*They (My parents) didn’t come. I hoped they would come but they didn’t.*

*I have no relatives to accept me. I killed my father. My mother died when I was 3. My sister never calls, and I don’t know how to contact her. She did not try to find me.*

*I will never get out of here. My brother and sister-in-law are my only relatives, but they are afraid that I will reoffend, so they refuse to pick me up.*

*They will not take me home even though I have been rehabilitated for years.*

### Guilt

All participants were both violent offenders and mentally ill patients. These identities may cause low self-appraisal and guilt, and further lead to depression. Some participants knew that they were both a patient and an offender, even though they were not fully conscious about their behaviors when they committed the crimes. Self-stigmatization haunted some participants, because they could not forgive themselves for murdering others. It was often the case that participants felt guilty about what they had done.

*I am a patient, and a criminal.*

*Indeed, I committed crimes, although I was out of my mind. But when these things happen, no one will listen to your explanation.*

*I look down upon myself. I killed people. I despise myself.*

*I feel so guilty. I am so sorry for the victim’s family.*

### Worthlessness

The participants reported that their lives at the hospital were worthless. The repetitive daily life was boring and was viewed as a waste of time. Some said that they would still be worthless and isolated from society after discharge. They worried that employers would not hire them due to their criminal history and that they would not fit into society again. They hoped that the hospital could provide some education and training programs to help them cope with stigmatization and maintain social relations and occupational skills.

*I feel like my life is meaningless.*

*I will be worthless even after discharge, people are going to mistreat me.*

*Companies will definitely kick me out. Nobody wants a ‘mental disabled’ person.*

*I will just stay at home when I am discharged.*

*Just ignore them (people who look down on me).*

### Mood and sleeping problems

#### Depression

Depression was common among participants in the hospital. Some reported consistent depressive status, accompanied by drowsiness, smoking cravings, body pains, etc. Some participants even wanted to hurt themselves because of depression and low mood. They attributed these symptoms to not adapting to life in the hospital, and homesickness. Participants suggested that they would not voluntarily seek assistance from medical staff as they barely had the chance to talk to them. However, most participants would agree to talk about their emotional turmoil if the doctors asked. Some participants chose to accept the reality as a way to escape from negative emotions.

*I want to hurt myself. I’m in a really bad mood.*

*I miss home. I want to see the outside world. I’m always upset, thinking about many staff.*

*I just want to smoke when I feel bad, or take a nap on the table.*

#### Sleep disorder

Sleep disorder was another common syndrome for the participants. Insomnia might persist for as long as one month. Most of the participants had reached out for help about their sleeping problems, including requests for dose adjustments, but suggested that they did not hear back. Some participants chose to talk about sleeping problems with their fellow patients. In addition, some participants were not satisfied with their sleeping time. They wanted one to two more hours of sleep.

*I was preoccupied by all kinds of thoughts. I couldn’t sleep well for nearly two months.*

*I talked to doctors (about sleeping problems), but it’s no use, it changes nothing.*

### Lack of freedom

Many participants thought that they were restricted and had no freedom, which upset them. Moreover, most participants were smokers, and restrictions on the amount of nicotine ingestion was one of their major concerns. Sometimes the nicotine limitation lead to strong suicidal ideation. Most of them complained about the strictly restrained smoking areas, and urged for the lift of this restriction.

*There is no freedom in this place. Wish I could be out soon to get some freedom.*

*The smoking area is too small, and it is in the lavatory. Snacks are not necessary, but not smoking is fatal.*

*Not smoking makes my head dizzy. Blood all goes up to my brain. I might as well hit the wall and die.*

*The money my family sent was all spent on cigarettes.*

### Lack of mental health intervention

None of the long-stay participants tried to seek psychological assistance. They received no psychological intervention, and knew very little about such services offered by the hospital. Psychological treatment resources were scarce in these forensic psychiatric hospitals. Only one participant mentioned that there were group interventions, but he did not understand the name nor purposes of the intervention. However, participants all expressed a need for psychological intervention. They wanted diverse types of psychological treatments, or one-to-one counselling, to improve emotion and relieve symptoms.

*I have never heard of these psychological treatments.*

*Certainly, I wish the hospital could offer some psychological treatment. It’s gonna be good for our conditions.*

## Discussion

This is the first in-depth individual interview study using a qualitative method on long stay mentally ill patients about their perception of long-term stay in forensic psychiatric hospitals in China. From the interview reports we identified seven themes reflected by long stay patients, including hopelessness, loneliness, worthlessness, mood and sleep disorders, lack of freedom, and lack of mental health intervention; which are clinically and legally instructive for the mental health service in forensic psychiatric hospitals.

The patients who participated in our interviews were mentally stable and had regained insight. This would have met the requirements for discharge at other general psychiatric hospitals, yet the patients here were kept in due to public safety considerations [30] without specific legal provisions about time limits. According to the *Criminal Procedure Law of the People’s Republic of China (2013 Amendment) Article 288*, “where risks to personal safety no longer exist and compulsory medical treatment no longer needs to be imposed, the facility should promptly recommend the person’s discharge and report to the people’s court imposing compulsory medical treatment to approve the discharge”. Prior to this law, patients were dealt with by the *Criminal Law Article 18:* “the government shall enforce hospitalization when necessary”, without mentioning about the discharge procedure. Nevertheless, neither of these two laws provide any practical guidelines on discharge assessment or follow-up treatment. This study also shows that patients only had a vague idea about discharge requirements and no access to the related legal information. The uncertainty about discharge may generate a sense of hopelessness. As the definition of recovery remains undefined in forensic settings [31], long stay in forensic hospitals is common and only a minority of patients can be discharged. The major reason for this is “risk”, although the re-offending rates are four and six times lower for mentally ill offenders treated at secure hospitals than in prisons [32]. This dilemma could remain until national strategies are taken. Unlike western countries such as the UK, where patients in high security hospitals would be referred to medium security hospitals and further discharged into the community, there is a lack of transfer system in China. Barriers include inadequate legislation, lack of lower security facilities and community psychiatric services. Moving forward, the government could legalize the discharge procedure of mentally ill offenders, implementing a practical procedure of violent risk assessment, rehabilitation standards, and post-discharge supervision management, and adding transfer facilities such as general psychiatric hospitals and wards to relieve the pressure of forensic psychiatric hospitals.

In addition, law assigns family members as patients’ guardians after discharge, which means that their decision plays a key role in whether patients could be discharged. However, as many patients had lost close family members or lost contact with them, complaints and distress occurred. Some patients believed that their family would not accept them after discharge, while others were disappointed about their family not mentioning any plans or arrangements for discharge during visits. In light of this, we suggest that the government should facilitate communication amongst patients and their families’, local communities, medical providers, and the forensic psychiatric hospitals as a way to share the patients’ families’ burden after discharge and increase the discharge rate of rehabilitated patients.

Loneliness is common in forensic hospital settings [21, 33]. Interviews with patients revealed that patients’ families often think of them as a liability and are not willing to visit them. Such rejection from families is associated with fear of the risk of re-offending. Many patients wished they could reunite with family in the hospital during holidays like Mid-Autumn or Spring Festival. Therefore, we suggest that families should be encouraged to be more supportive and to visit during holiday seasons. Hospitals could hold some activities during family visits or allow online video-chatting opportunities, to reduce patient loneliness. Personal relationships are often plain in the hospital; patients do not communicate with each other very much. The hospitals could organize more social activities, both among the patients themselves and between patients and other social groups. For example, hospitals could invite some volunteer workers to interact with the patients. These activities could help reduce loneliness, build social support, and restore patients’ normal social functioning.

Our findings indicated that most participants suffered from depression, which may also be accompanied by physical and emotional distress. However, they never sought medical assistance for such distress, and did not know how to deal with negative emotions. Extreme incidents such as self-harm were reported. Therefore, we suggest that medical staff pay more attention to the observation and assessment of patient’s mood and sleep behaviors.

Importantly, this study showed that almost all participants had smoking issues. Some patients started smoking to pass time or to deal with bad moods, because their daily life was dull and uninteresting. Many patients stated that smoking restrictions should be released. Previous quantitative research also indicated high prevalence of smoking in this population and a total smoking ban may be impossible to implement without serious negative consequences [16, 23]. Smoking restrictions had caused suicidal thoughts among some participants. Therefore, the hospital should initiate interventions including smoking cessation for addictive smokers.

Our study demonstrated that forensic psychiatric hospitals are poorly prepared for psychological intervention from which the patients may benefit [34]. All participants expressed some intentions of receiving psychological counselling to cope with emotion, sleep problems and suicidal thoughts. This may also help them to gain a better understanding about their condition. Previous research has criticized the deficiency of China’s mental health services [35]. Psychological intervention is not usually available in general hospitals or psychiatric hospitals [36, 37], let alone in forensic psychiatric hospitals. We suggest that hospitals should provide better psychological interventions. Lack of communication between patients and medical staff could be due to the limited quantity of staff, but may also be because patients are intimidated by potential consequences of reaching out. Patients worried that telling the truth to the doctors may affect their discharge progress. As a result, one-to-one psychological intervention plans should be scheduled by therapists to establish reliable doctor-patient relationships.

In addition to strong stereotyping by the public [38], this study also suggested that self-reproach and self-stigmatization exists among long stay patients, which is in accordance with previous qualitative studies [24, 39]. They expressed concerns about other people’s opinions on them, and how to deal with such stigmatization and relationships. Therefore, it is advised that the psychological intervention provided at hospitals should aim to deal with self-reproach and self-stigmatization in order to raise patients’ sense of value. Participants also complained about the monotonous and restricted life in forensic psychiatric hospitals. Lack of sensational news in life can easily lead to negative feelings of unworthiness, boredom, frustration, and depression, which may negatively affect their recovery. As suggested by previous qualitative evidence that patients’ recovery in forensic hospitals may benefit from activities with employment prospects [21, 25, 26], we suggested that the hospitals should provide recovery-oriented programmes by increasing recreational activities, working opportunities and workshops on occupational and interpersonal skills.

### Limitations

Our findings should be interpreted with caution. Firstly, as the sample was recruited from one hospital, it may not be representative of all forensic psychiatric patients in China. However, hospital settings tend to be similar, so our findings are generalizable to some extent. Secondly, Our sample had a higher proportion of male patients, which may not reflect gender-specific characteristics. Thirdly, we did not use formal instruments to assess the insight of the participants. The definition of “good insight” was based on the participant’s psychiatrist report.

## Conclusion

Despite their criminal-related identities, quality of life among long stay mentally disordered offenders deserves care and attention. This study defines seven themes, reflecting their experiences and needs, which may serve as future clinical clues to mental health recovery among the population. There are some important implications for management of the research: prompting for the legalization of discharge assessments; enhancing daily observation and assessment on patient’s mood and sleep, introducing diverse types of psychological therapies to deal with patient’s loneliness, self-reproach, negative emotion management, and unworthiness; providing cessation interventions for addictive smokers. Future research should attempt to conduct surveys on smoking habits and addiction, and effective interventions to improve the quality of life in forensic settings.

## Additional file

Additional file 1: Interview schedule (translated from Chinese). (DOCX 16 kb)

## Data Availability

The datasets used and/or analysed during the current study are available from the corresponding author on reasonable request.
