# Supplementary material for: The experience of long-stay patients in a forensic psychiatric hospital in China: a qualitative study
Source: BMC Health Serv Res. 2019 Sep 2;19:617. doi: 10.1186/s12913-019-4458-6 (PMC6721342; doi:10.1186/s12913-019-4458-6)
Supplement: Supplementary file 1 — Interview schedule (translated from Chinese). (DOCX 16 kb) [file 12913_2019_4458_MOESM1_ESM.docx]

**Interview schedule (translated from Chinese)**

1. Needs for activities, work and courses

How do you pass the time during your stay in the hospital?

How do you feel about the activities you undertake in the hospital?

How do you view the paid work that the hospital has arranged for you? Do you have any other comments about the work?

How do you feel about the courses in the hospital?

2. Willingness to leave the hospital

How long have you been hospitalized?

Have you ever thought about discharging from the hospital?

Have you made any effort to do this?

Why did not you leave the hospital yet? What are the main reasons (obstacles)?

3. Knowledge about legal rights and interests

What conditions do you think are necessary to be discharged?

Do you know how to request a discharge?

What do you think about the regulations about the length of stay, as neither criminal law nor criminal procedure law has a specific definition regarding it?

Are the current laws reasonable to you? What comments do you have?

4. Discharge plan

Have you ever thought about hospitalization for a lifetime? What are your plans?

If you are discharged from the hospital, what plans do you have for after leaving?

Have you ever worried that you might re-offend, or conduct uncontrollable behaviours?

What can do you to avoid or reduce these behaviours?

How will you improve your relationship with others?

5. Viewpoints about the case

What the reason was you hospitalized?

How do you feel about the case now?

Do you think this hospitalization was reasonable?

Has your attitude towards hospitalization changed during the stay? How did it change? What is the cause of the change?

6. Insight

Do you think you have a mental health issue?

How do you view your current physical and mental condition?

How do you feel about taking medicine for a long time?

7. Life satisfaction

Are you satisfied with your current life?

What makes you feel satisfied/ what is not satisfied?

Is there anything that can be improved, such as management, health services?

8. Interpersonal relationship

How do you usually keep in touch with your family?

How is your relationship with other patients?

How is your relationship with your doctor?

9. Psychological health

Have you ever felt any emotional distress during your stay?

How did you deal with it?

Have you ever asked for help from health care staff?

Have you ever hurt yourself?

Have you ever wanted to die? What was the reason and when?

Have you received psychological counselling or psychological treatment? Do you think you need it?
